# Molecular Modeling of the Deamidation Reaction in Solution: A Theoretical–Computational Study

**DOI:** 10.1021/acs.jpcb.3c04662

**Published:** 2023-10-30

**Authors:** Maria Laura De Sciscio, Alessandro Nicola Nardi, Fabio Centola, Mara Rossi, Enrico Guarnera, Marco D’Abramo

**Affiliations:** † Department of Chemistry, University of Rome, Sapienza, P.le A. Moro 5, 00185 Rome, Italy; ‡ Global Analytical Development, Merck Serono S.p.A., 00012 Guidonia Montecelio, Italy; § Antibody Discovery and Protein Engineering, Merck Healthcare KGaA, 64293 Darmstadt, Germany

## Abstract

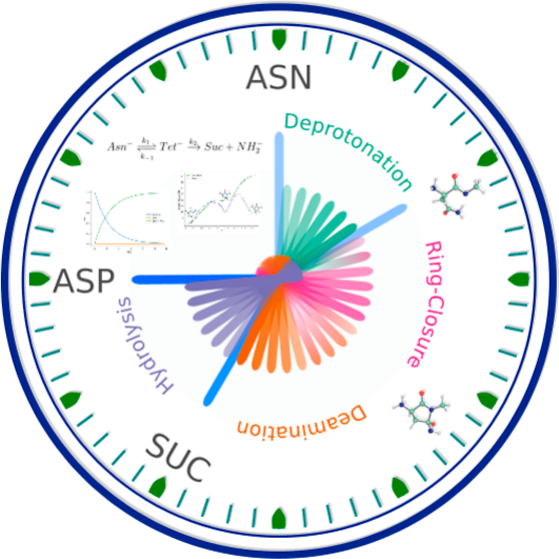

In this work, a theoretical–computational method is applied to study the deamidation reaction, a critical post-translational modification in proteins, using a simple model molecule in solution. The method allows one to comprehensively address the environmental effect, thereby enabling one to accurately derive the kinetic rate constants for the three main steps of the deamidation process. The results presented, in rather good agreement with the available experimental data, underline the necessity for a rigorous treatment of environmental factors and a precise kinetic model to correctly assess the overall kinetics of the deamidation reaction.

## 1 Introduction

Amino acids bearing an amide group on the side chain, i.e., asparagine (Asn) and glutamine (Gln), can undergo spontaneous nonenzymatic deamidation, yielding aspartic acid (Asp) and glutamic acid (Glu), respectively. This post-translational modification (PTM) entails the conversion of a neutral side-chain to a negatively charged one, i.e., Asp and Glu acidic functions are deprotonated at physiological pH, and it represents a common pathway of chemical degradation of peptides and proteins.^1,2^ Among these two residues, Gln deamidation is significantly slower than Asn one and has been detected mainly in long-lived proteins.^3,4^ On the other hand, deamidation of Asn residues, due to its high rate and frequency in proteins, is involved in several pathological events,^5^ and the increasing content of deamidated products in proteins is related to aging conditions and possible loss of biological activities.^6,7^

Furthermore, deamidation is a common PTM in biopharmaceutical proteins that can affect the stability, biological activity, and efficacy of the product, making it an important critical quality attribute (CQA) in drug development.^8,9^ The rate of deamidation reactions is influenced by various factors, such as protein structure, temperature, and pH.^9^ For instance, deamidation rates are greatly enriched in asparagine if followed by glycine and, to a lesser extent, if followed by serine.^10^

**Figure 1:**
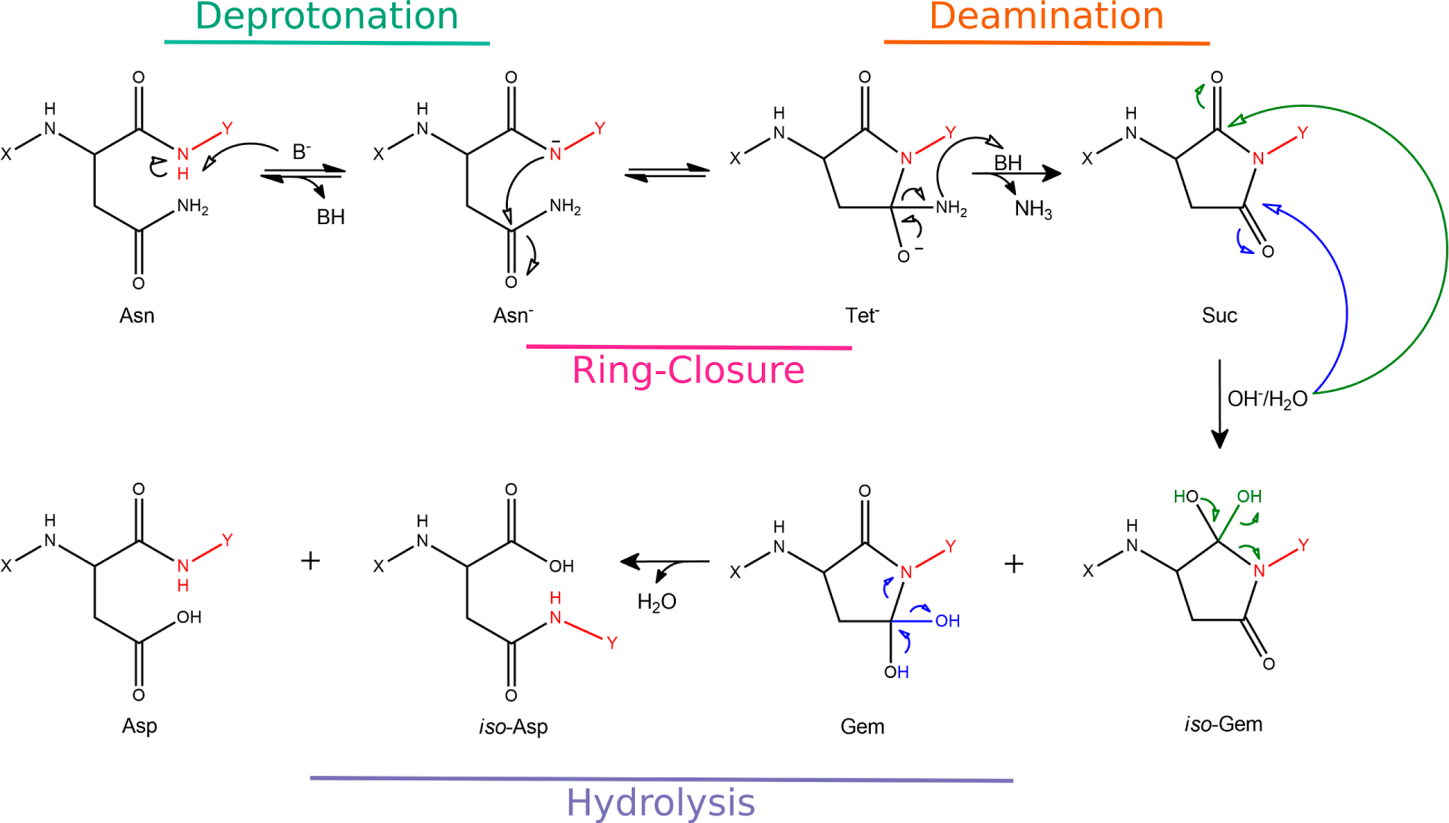
Deamidation mechanism. The reaction begins with the deprotonation of the Asn, resulting in Asn^–^ formation, which leads to the intramolecular ring closure by attacking the Asn carbon atom of the amide side-chain. The metastable tetrahedral intermediate (Tet^–^) loses the negatively charged NH_2_ group, leading to a succinimide intermediate (Suc). Then, two hydrolytic paths are possible: one leads to Asp (blue) and the other to *iso*-Asp (green), via the gemdiol (Gem) or *iso*-gemdiol (*iso*-Gem) intermediate formation. Atoms in red represent *n* + 1 amino acid, while X and Y are the C-terminal residues and the N-terminal one from the Cα atom, respectively; in this work, X = H and Y = CH_3_.

Although the reaction mainly alters the amino acid side chain, an essential role is played by the C-carboxyl residue (residue *n* + 1) at physiological pH. In fact, according to the literature,^11^ deamidation proceeds through succinimide formation via a three-step reaction. As reported in Figure 1, the process starts with the deprotonation of the nitrogen atom belonging to the (*n* + 1) residue, and it is followed by a spontaneous attack of the latter atom, now negatively charged, on the Asn side-chain amide carbon atom, leading to the formation of a cyclic tetrahedral intermediate (Tet^–^). The metastable product naturally evolves into the succinimide intermediate (Suc), which is an experimentally characterized product. Thereafter, the hydrolysis of both sides of the imide can occur, leading to Asp or *iso*-Asp.

Succinimide formation (ring closure and deamination stages) and, in particular, the conversion of Tet^–^ to imide, entailing deamination, were identified as the rate-determining step (RDS) at pH = 6–8.^11^ Therefore, the molecular features of these stages directly correlate with the overall deamidation rate.

In neutral-to-basic conditions, deamidation rates are influenced by several factors linked to the different reaction stages: (i) deprotonation is affected by acid–base equilibrium and the type of residue in position *n* + 1, upon which p*K*_a_ depends^12^ (ii) ring closure and deamination stages are strongly affected by the torsional angles of Asn and of the *n* + 1 residue,^10,13,14^ as well as by any factors able to modify the side-chain amide carbon atom and the *n* + 1 nitrogen electrophilicity (iii) hydrolysis stage mainly depends on water molecules/hydroxide ion proximity to imide hydrolytic sites.

Several computational studies focused on deamidation in proteins used to evaluate the reaction through classical descriptors—such as Asn averaged Solvent Accessible Surface Area (SASA), C_γ_–N_*n*+1_ distance, root means square fluctuations (RMSF), backbone and side-chain dihedrals (e.g., χ and ψ), and H-bond analysis—by means of classical molecular dynamics simulation and (static) structure-based approaches.^1,10,13−19^ The limited quantum mechanics/molecular mechanics (QM/MM) works often approximate the deamidation reaction to a single-stage Asn → Suc reaction,^13,14^ which may affect the accuracy of the prediction.^14^ Furthermore, for computational reasons, more approximate QM semiempirical methods are usually employed to describe the reaction. On the other hand, QM works—focused on mechanistic investigations—do not include an explicit treatment of the solvent, which leads to an overestimation of the energy barriers of the reaction (higher than 30–40 kcal/mol),^20−22^ not in a good agreement with the experimental estimates of reaction half-life times and Arrhenius activation energies (≃20–25 kcal/mol).^2,23^

**Figure 2:**
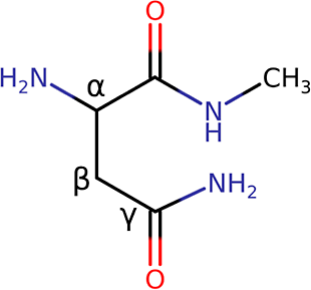
2D representation of the model system, i.e., NH_2_–Asn-NMe, used in this study.

Although several efforts were made to understand the deamidation reaction features, the structural determinants allowing the correct prediction of Asn residues prone to deamidate are not yet completely understood. This work aims to give new insights into the deamidation mechanism by means of an accurate theoretical–computational QM/MM approach that can provide a quantitative estimate of the kinetics of the deamidation reaction in a simple model system (NH_2_–Asn-NMe, Figure 2).

Hence, we reconstruct the free energy barriers of the three steps leading to the formation of the Asp—i.e., ring-closure, deamination, and hydrolysis stages—in a fully solvated condition. With such an approach, we were able to model the kinetics and, therefore, predict the overall reaction behavior, which is in rather good agreement with the available experimental data on similar systems.

## 2 Theory

### 2.1 Perturbed Matrix Method

In this work, we applied the Perturbed Matrix Method (PMM)—a theoretical-computational quantum mechanics/molecular mechanics (QM/MM) approach—to study the deamidation reaction. As the PMM theory has been fully detailed in previous papers,^24−29^ here we provide only the main features and the relevant equations used in this work. According to the PMM scheme and similar to other QM/MM approaches, the system is subdivided into two regions, one composed of the atoms directly involved in the process of interest and known as the Quantum Center (QC), and the complementary part, which constitutes the environment. The former is treated by means of quantum-chemical calculations, while the latter, acting as the perturbing environment, is described by classical molecular dynamics (MD) simulations. Unlike “on the fly” QM/MM methods, within the PMM approach, the effect of the environment is added a posteriori, allowing for the inclusion of the statistical fluctuation of the QC environment at a reasonable computational cost. In the PMM scheme, for each QC nuclear configuration, the electronic Hamiltonian  of the QC can be expressed as the sum of the isolated QC electronic Hamiltonian, i.e., the unperturbed gas-phase electronic Hamiltonian of the QC, , and the perturbation operator, , as reported in eq 1.
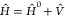
1

The corresponding electronic Hamiltonian matrix elements , expressed in the basis set of the unperturbed electronic QC eigenstates Φ_*l*_^0^ and eigenvalues υ_*l*_^0^, can be obtained as
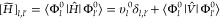
2

The perturbation operator, describing QC-environment interaction, can be obtained via a multipolar expansion centered in the QC center of mass (**r**_**0**_)
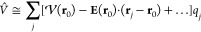
3where (**r**_**0**_) and **E**(**r**_**0**_) are the perturbing electric potential and the electric field produced by the environment, respectively; the *j* index runs over all QC electrons and nuclei, *q*_*j*_ is the charge of *j*-th particle, and **r**_*j*_ is the associated coordinates. The multipolar expansion, reported in eq 3, is explicitly treated up to the dipolar term for the off-diagonal elements of the electric Hamiltonian matrix. The diagonal elements of the  matrix are calculated within the atom-based expansion PMM level of theory, according to which  is obtained as follows
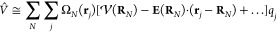
4where *N* refers to all QC atoms, Ω_*N*_ is a step function that is null outside and unitary inside the *N*-th atomic region, and **R**_*N*_ is the *N*-th nucleus position. Hence, through the diagonalization of , at each MD frame, a trajectory of QC perturbed electronic eigenstates and eigenvalues is obtained. When the calculation is performed along the reaction coordinate (ξ), as in the present work, the Helmholtz free energy of the reaction can be calculated as a function of such a coordinate, as previously reported.^25,30−32^ Thus, the perturbed free energy change (Δ*A*), due to the movement from an initial position (i) to a subsequent point (*i* + 1), along the reaction coordinate is
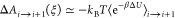
5where Δ*U* is the perturbed energy change, *k*_B_ is the Boltzmann’s constant, and β = 1/*k*_B_*T*. The sum of each free energy variation leads to the Δ*A*_tot_.

### 2.2 Reaction Kinetics

A general first-order reaction, starting from a reactant species (R) and spontaneously evolving to a different compound, the product (P), can be generally expressed as
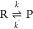
6

When *k* and *k*′ are of the same order of magnitude, the reaction rate , defined as the time (*t*) rate of the increase in the reaction extent (*x*), or the speed at which reaction occurs for the law of mass action, is
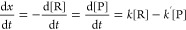
7

Each of the possible reaction paths, i.e., the forward and reverse ones, reasonably goes through a unique transition state (TS), such that the reaction can be schematized for the forward path as
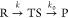
8

In the case of *k* ≪ *k*_0_, the steady state condition for TS and an equilibrium within the tiny TS region, which implies that the rate constants of the inward and outward fluxes are equal, can be assumed. Thus, the TS population has the same probability of falling into R or P, and the rate constant *k*_0_ times  is the TS rate constant, i.e., *k*_0_^′^ ≃ *k*_0_/2. It can therefore be written
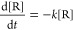
9and
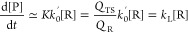
10where *K* = *k/k*_0_^′^, while *Q*_TS_ and *Q*_R_ are the canonical distribution functions of the TS and reactant, respectively. As a reaction can be followed along a reaction coordinate, defined as ξ, it is possible to express and obtain the reactant and transition state partition functions within ξ ensembles. Finally, assuming a homogeneous density in the TS region, the Landau kinetic constant can be written as
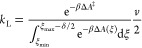
11where the integral limits (ξ_min_, ξ_max_ – δ/2) define the reactant ensemble ξ area—from the energy minimum, i.e., the reactant, to the TS immediately preceding point—and Δ*A*(ξ) is the Landau free energy variation along ξ, while Δ*A*^‡^ is the free energy variation between the transition state and the reactant. β = 1/*k*_B_*T*, and *v* represents the mean velocity at which TS transits toward the product.

A more detailed explanation of the mathematical derivations of eqs 10 and 11, as well as the meaning and calculation of Landau free energy from the Helmholtz one, can be found in our previous work.^30^

When the reactant area shape is fully quadratic and the TS traversing velocity (*v*) is approximated to a normal mode fluctuation, eq 11 becomes the well-known Eyring equation
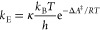
12where *k*_B_ is the Boltzmann’s constant, *T* the temperature, *R* the ideal gas constant, and κ the transmission coefficient.

### 2.3 Deamidation Kinetic Model

A full description of the deamidation reaction and associated kinetic rate constants, considering only one hydrolysis path (i.e., the blue one in Figure 1), is as follows
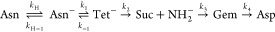
13

To simplify the complex resulting kinetic scheme, we applied two approximations: (i) the first stage, that is, deprotonation, can be considered a fast pre-equilibrium with a high *K*_Eq_ = *k*_H_/*k*_H–1_, i.e., product formation is favored; (ii) *k*_3_ and *k*_4_ are far higher, i.e., hydrolysis is faster, than *k*_1_ and *k*_2_.

Accordingly, the deamidation reaction scheme becomes
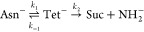
14

The associated differential equations are
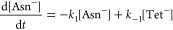
15
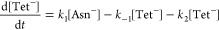
16
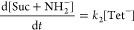
17

The exact solutions of these equations,^33^ taking both [Tet^–^] and [Suc + NH_2_^–^] to be equal to 0 and [Asn^–^] equal to 1 at *t* = 0, are
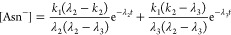
18
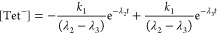
19
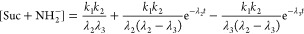
20where the eigenvalues λ_2_ and λ_3_ are obtained from the kinetic matrix determinant, defined as follows
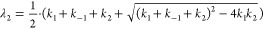
21
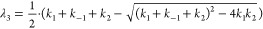
22

## 3 Methods

The estimation of the free energy profiles, kinetic rate constants, and deamidation half-times required several operative steps. For each stage of the deamidation reaction (Figure 1), starting after the deprotonation, the calculated electronic properties in vacuum and the effect of the perturbing environment, evaluated by means of classical molecular dynamics simulations, were combined (via eq 1) to obtain the free energy variation along the selected reaction coordinate. The free energy variations were always obtained by means of the PMM, as shown in eq 5. The rate constants associated with forward and backward reactions were calculated through eqs 11 and 12. The evaluation of the profile shape and rate constants allowed us to choose the most representative kinetic scheme for the deamidation reaction. Using eqs 18–20, the populations of the involved species versus time and the corresponding half-life (τ_1/2_) were estimated. The block-averaging procedure was applied for the estimation of Δ*A* statistical error, while *k*_L_, *k*_E_, and τ_1/2_ were evaluated by recalculation of the values according to Δ*A* statistical fluctuations. The system used to study the reaction of interest is composed of an N-terminal Asn where the carboxylic function is capped with a methyl amine (NH_2_–Asn-NMe), as can be seen in Figure 2.

### 3.1 Quantum-Chemical Calculations

The QM calculations were performed with the Gaussian 16 software^34^ using density functional theory (DFT) and time-dependent density functional theory (TD-DFT) with the B3LYP functional and 6-311++G(2d,2p) as the basis set. The starting geometry was obtained by a structural analysis of the MD simulation of Ace-Gly-Asn-Gly-Gly-NMe (details are given in the following section). For each reaction step, the reactant was first optimized without constraints and subsequently a relaxed scan was run to achieve the energy profile; the last point, i.e., the product, was optimized in the absence of restraints, while TS search was performed using QST2 or QST3 routine and checked for the correct characteristic imaginary frequency. Finally, the IRC was calculated to verify the reactant and product associated with the TS as well as the path to them. Once the profile was obtained, for every optimized geometry (to use in PMM calculation), gas-phase properties, such as electronic energies, dipoles, and ESP charges, were obtained for the ground state and the first three excited states. The QC is made up of NH_2_–Asn-NMe for the first two investigated reaction stages (i.e., Ring-Closure and Deamination, Figure 1), while for the hydrolytic steps were also added two water molecules, in the case of the water-mediated mechanism, and a hydroxide ion with an additional water molecule to stabilize the OH^–^ anionic charge in vacuum for the alkaline catalysis. The following distances were chosen as reaction coordinates (ξ) for the corresponding reaction steps:

(i) N_*n*+1_–C_γ_ for the ring-closure process (Asn^–^ → Tet^–^).
(ii) C_γ_–N_γ_ for deamination stage (Tet^–^ → Suc + NH _2_^–^).
(iii) C_γ_–O of OH^–^/H_2_O in the first hydrolysis stage (Suc → Gem).
(iv-1) difference of N_*n*+1_–C_γ_ and N_*n*+1_–H of H_2_O for the second hydrolysis stage within the water-mediated mechanism (Gem → Asp).
(iv-2) N_*n*+1_–C_γ_ for the second hydrolysis stage within the alkaline catalysis mechanism (Gem → Int).
(v) N_*n*+1_–H of H_2_O for the third hydrolysis stage according to the alkaline catalysis mechanism (Int → Asp).

### 3.2 Molecular Dynamics Simulations

Molecular dynamics simulations were performed using Gromacs-2021^35^ and Q-FORCE/OPLS as the force field.^36−38^ The Lennard-Jones parameters are derived from the OPLS FF, while all the bonded parameters for the solute molecules are calculated by ab initio calculations, following the procedure described by Sami and co-workers.^36^ Atomic charges were estimated by the ESP procedure.^39,40^ The QC, coinciding with the solute in our work, was placed in a cubic simulation box large enough to avoid boundary effects, which was then filled with water molecules (using the TIP3P water model^41^) and neutralizing ions (Na^+^). For the solvent molecules, OPLSAA force field parameters were used.^38^ For all the systems, the production runs were performed in the *NVT* ensemble after an energy minimization of the system with the steepest descent algorithm and short equilibration steps, in which the box size was adjusted to reproduce the experimental water density.^42^ The temperature was kept constant (at 300 K) by the velocity rescaling algorithm,^43^ with a τ_T_ of 0.01 ps. Simulations, lasting at least 150 ns, were carried out with a time step of 2 fs; long–range interactions were evaluated with the particle mesh Ewald method,^44,45^ and the Verlet cutoff scheme was used to generate the neighbor lists.^46^ The cutoff for electrostatic and van der Waals interactions was 1.1 nm. To ensure the calculation of Δ*A*(ξ), i.e., the free energy variation at fixed values of the reaction coordinate, the QM optimized geometry was used as the starting configuration for MD simulation, constraining it by freezing its atomic coordinates or adding a harmonic potential to the distance of interest. In particular, for the Asn^–^ simulation, the solute atomic coordinates were fixed, while in all the other cases, the distance between the atoms involved in the bond formation or cleavage was kept fixed. An additional simulation of the capped tetrapeptide Ace–Gly–Asn–Gly–Gly–NMe^11^ was performed to obtain the initial conformation that is used for the QM calculations in our model system. A cluster analysis,^47^ using a cutoff of 0.15 nm on the 500 ns simulation, allowed us to extract the most sampled Asn–Gly reactive-like conformation, as defined by χ dihedral (C–C_α_–C_β_–C_γ_) values and C_γ_–N_*n*+1_ distance of ∼70° and 3.4 Å, respectively.^1,10,13,14,19^

## 4 Results and Discussion

The deamidation reaction, shown in Figure 1, was studied in a fully solvated system. Such a reaction consists of four main consecutive steps: deprotonation, ring closure, deamination, and hydrolysis. As Asn deprotonation is usually very fast, we disregarded this step in the calculation of the reaction kinetics.

### 4.1 Succinimide Formation in Solution

**Figure 3:**
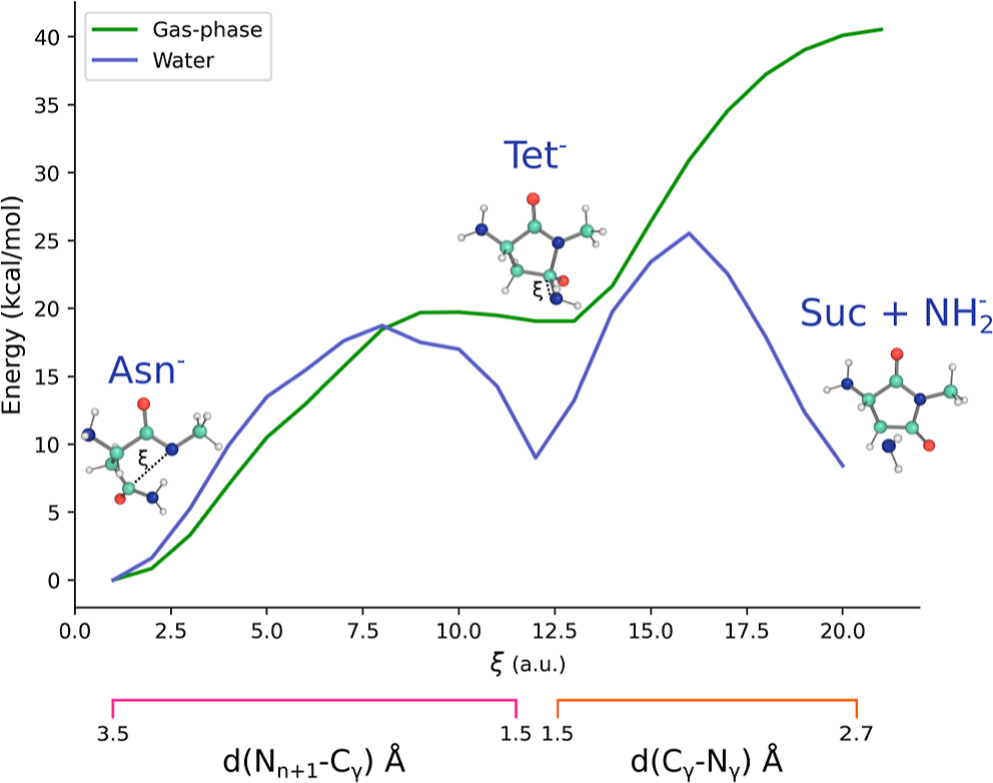
Two-step succinimide formation (free) energy pathway in the gas phase and in solution. The horizontal axis reaction coordinate ξ (a.u.) in the range 1–12 represents the C_γ_–N_*n*+1_ distance, while in the deamination stage it is C_γ_–N_γ_ (ξ = 12–20 a.u.). At left, middle, and right are reported the graphical 2D representation of Asn^–^, Tet^–^, and Suc + NH _2_^–^, highlighting the reaction coordinate ξ used in the two reaction steps.

According to the proposed deamidation mechanism,^11^ once Asn is deprotonated, succinimide intermediate is obtained through the ring closure and the NH_2_ expulsion as a leaving group via Tet^–^ intermediate. Hence, for the Asn^–^→ Tet^–^ step, the C_γ_–N_*n*+1_ distance was selected as the reaction coordinate (ξ) to describe the ring closure process, while for the deamination step, the reaction coordinate is represented by the C_γ_–N_γ_ bond length (see Figure 3).

The initial reactant conformation was obtained from an additional MD simulation of the Ace–Gly–Asn–Gly–Gly–NHMe tetrapeptide,^11^ taking the central structure of the most sampled cluster. The free energy profile of succinimide formation was obtained according to the PMM scheme discussed in the Theory and Methods section, with an estimated error on Δ*A*(ξ) < 0.1%.

As can be seen in Figure 3, the free energy path associated with the succinimide formation clearly represents a two-step reaction, where the first relative minimum (ξ ≈ 12 a.u.) is associated with the metastable product Tet^–^. In the second step, the crossing of a similar free energy barrier leads to the succinimide intermediate (ξ ≈ 20 a.u.). The effect of the solvent is to stabilize the intermediates, while the transition state for both reactions is only slightly affected by the presence of water molecules.

**Figure 4:**
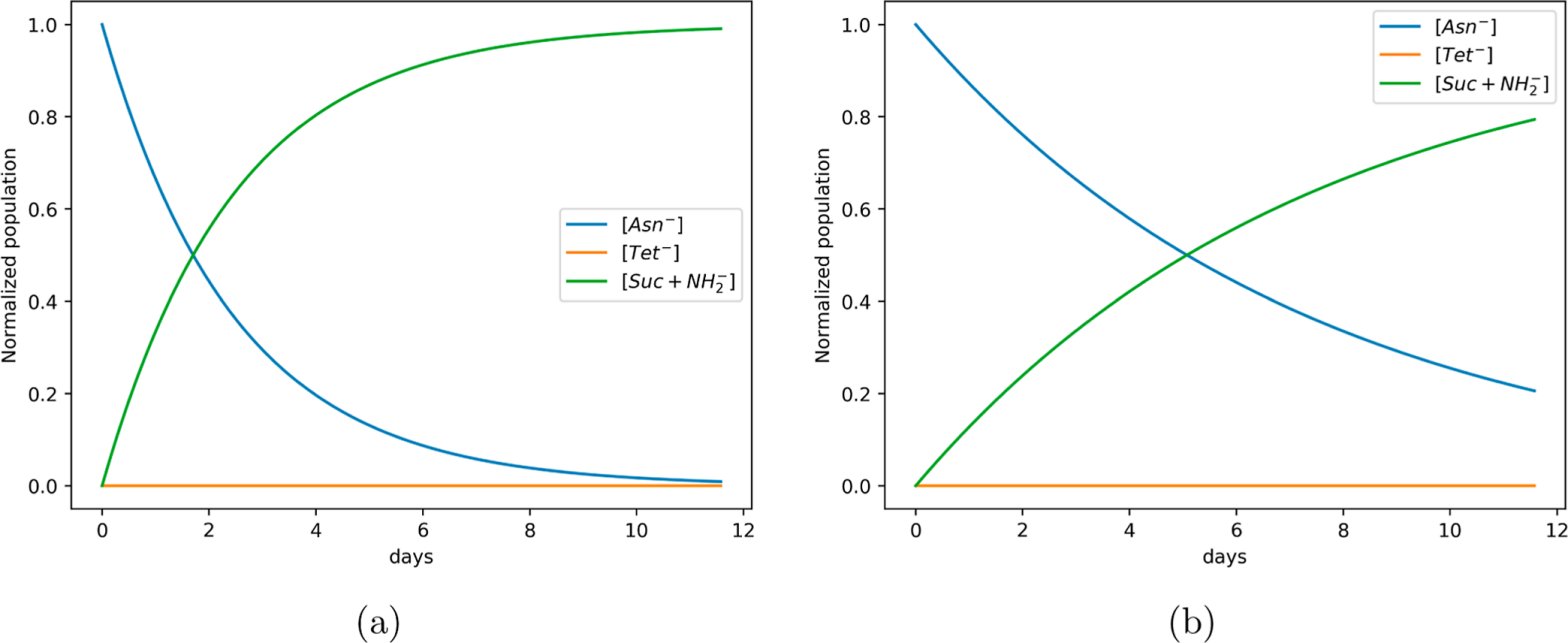
Time evolution of Asn^-^, Tet^-^, and Suc + NH_2_^-^ normalized populations within the kinetic model used in the present work (eqs 18–20) with (a) Landau kinetic constants: *k*_L1_ = 7.0 × 10^–1^, *k*_L–1_ = 4.3 × 10^6^, and *k*_L2_ = 28.9 s^–1^; (b) Eyring kinetic constants: *k*_E1_ = 1.40 × 10^–1^, *k*_E–1_ = 5.12 × 10^5^, and *k*_E2_ = 5.79 s^–1^.

It is worth noting that in this model system,  is equal to 18.7 kcal/mol, while  is 16.5 kcal/mol, in rather good agreement with a previous computational study.^48^ From the overall free energy profile of succinimide formation, it is possible to evaluate the kinetic rate constants of the process. From Figure 3, it comes out that , meaning that when the tetrahedral intermediate is formed, the reaction is able to go back to the reactant, affecting the overall kinetics of the process. Hence, to estimate the time required for succinimide formation, we apply the kinetic scheme described in the Deamidation kinetic model section. The resulting population profiles of the species involved in the reaction versus time are reported in Figure 4.

The calculated τ_1/2_ of succinimide formation—the time at which half of the product is formed—is, at 300 K, 1.7, or 5.1 days using Landau or Eyring equations, respectively. It is worth noting that, considering the reaction leading to the formation of the Tet^-^ intermediate as irreversible, remarkable differences in the reactions’ kinetics arise (see Figure S1). Therefore, for proper treatment of the reaction kinetics, it is mandatory to identify the correct kinetic scheme.

### 4.2 Succinimide Hydrolysis: Water-Mediated vs Hydroxide Ion Catalysis

Due to the imidic nature of the species of interest, whose reactivity is known to be slightly lower than the corresponding amide and, of particular interest here, more resistant to hydrolysis, two hydrolysis mechanisms were taken into account: the alkaline catalysis and the water-mediated mechanism. A direct water attack on the poor electrophilic carbon was considered inappropriate, while acidic hydrolysis was not included because at pH < 5 deamidation proceeds via a direct reaction, which does not involve the formation of the succinimide intermediate.^2,11^

**Figure 5:**
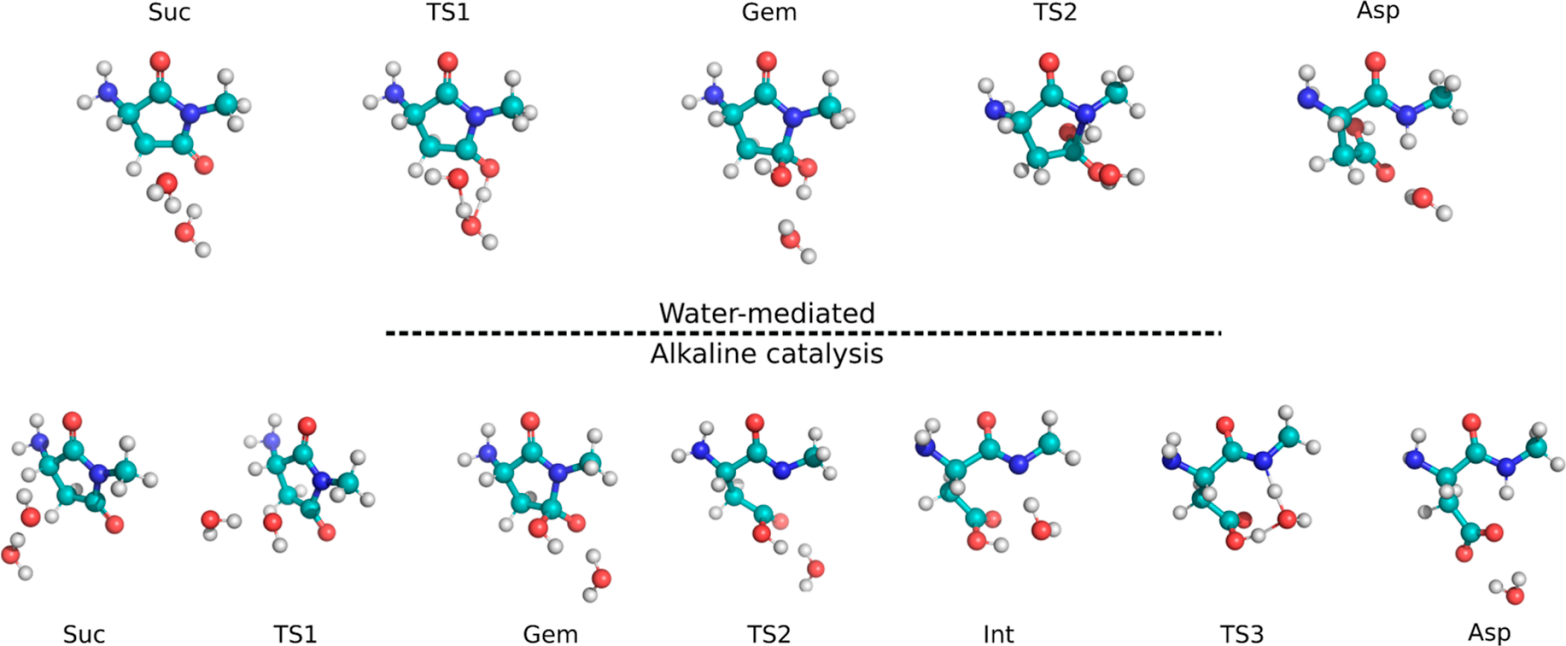
Succinimide hydrolysis mechanisms: water-mediated (upper panel) and alkaline catalysis (lower panel). Optimized structures of reactants, transition states (TSs), intermediates, and products are reported for each mechanism.

Therefore, we evaluated the two possible mechanisms for Asp generation from succinimide in the gas phase, while only the RDS for this stage has been studied in solution. In the water-mediated mechanism, two water molecules were included in the QM calculations,^20,49^ where one acts as a nucleophile while the other activates both the electrophilic and nucleophilic species, catalyzing the reaction. The second water molecule is, in fact, essential to form the active nucleophilic species, the hydroxide ion, obtained by an exchange of protons between the three oxygen atoms (i.e., the attacking oxygen transfers a proton to the second water molecule, which, acting as a proton shuttle, makes the attacked carbonyl an alcohol; Figure 5, upper panel).

When the gemdiol (Gem) intermediate is formed, the reaction proceeds through the concerted five-term ring opening and the N protonation by the second water molecules (the bidimensional potential energy surface is shown in Figure S2), which is restored via a proton transfer from one of the side chain hydroxyls, leading to the final product (Figure 5, upper panel).

On the other hand, alkaline catalysis shows lower energy barriers than the former process, reported in Table 1, as the stronger nucleophile easily attacks the inactive imidic carbon. In this way, the anionic form of Gem is produced, which subsequently leads to the ring opening. The final, fast protonation of the nitrogen atom in position *n* + 1 by a water molecule leads to the deamidated product Asp in its carboxylated form. The reactant, transition states, and products are reported in Figure 5. From that Figure, it can be seen that alkaline catalysis requires an additional step with respect to the water-mediated case but appears mainly barrier-less. The gas-phase energy barriers for both hydrolysis mechanisms and the corresponding rate constants are reported in Table 1.

**Table 1:** Hydrolysis Energy Barriers and Kinetic Rate Constants in the Gas phase (Calculated Using eq 12)

	reaction step	Δ*E*^‡^ (kcal/mol)	*k* (s^–^^1^)
water-mediated	Suc → Gem	37.2	5.1 × 10^–^^15^
	Gem → Asp	19.7	2.6 × 10^–^^2^
alkaline catalysis	Suc → Gem	2.1	1.7 × 10^4^^a^
	Gem → Int	7.5	1.4 × 10^7^
	Int → Asp	0.38	3.2 × 10^12^

a Second order rate constant, pH = 7.

**Figure 6:**
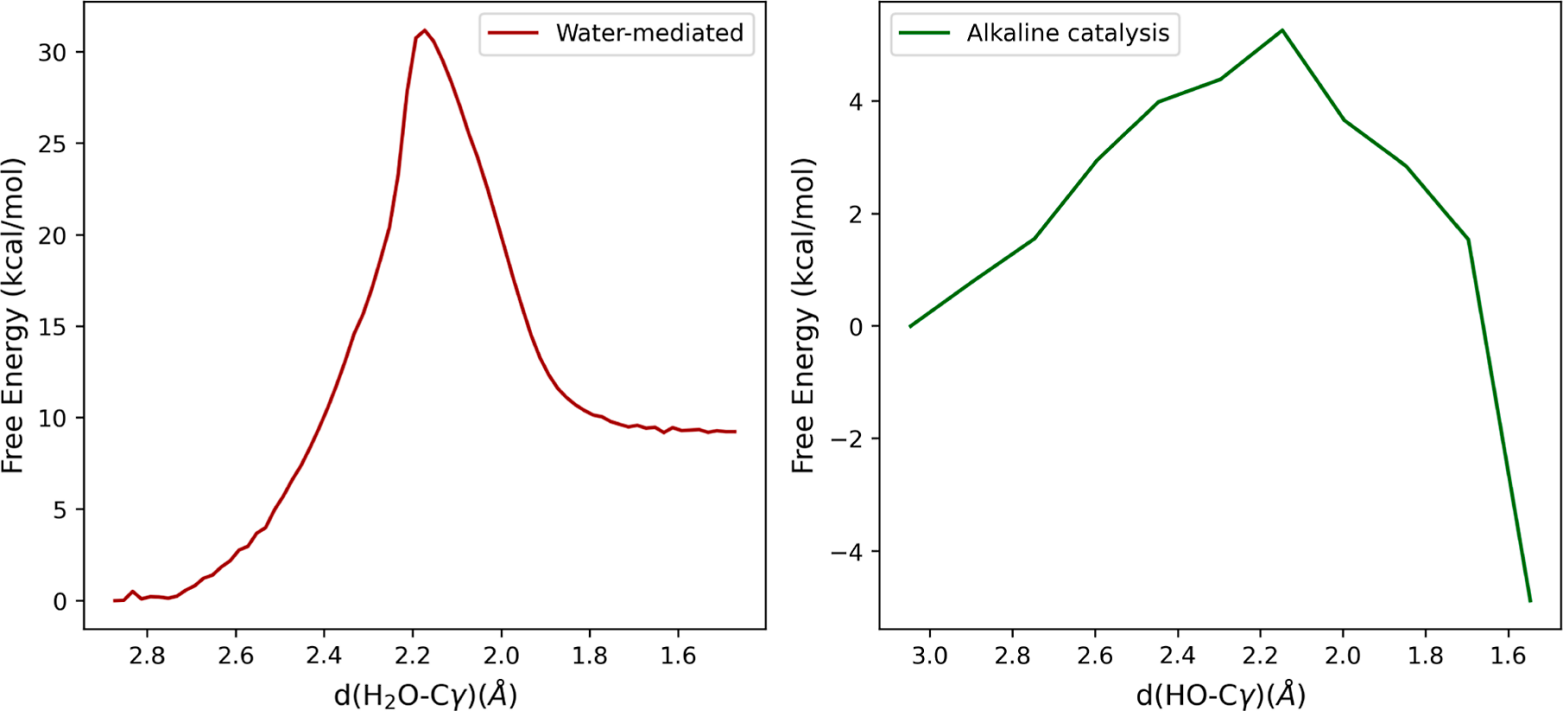
Free energy of the RDS of succinimide hydrolysis considering the water-mediated mechanism (left) and alkaline catalysis (right).

In the water-mediated mechanism, water attack requires a larger activation energy, in agreement with literature data,^20^ while in alkaline catalysis, the ring-opening step limits the reaction kinetics (Gem → Int). However, from a kinetic point of view, these processes are bimolecular, and thus, the concentration of the other reactants needs to be taken into account. In water, the dependency of the rate constant on its concentration can be neglected, while the alkaline catalysis is affected by the concentration of the nucleophilic species (i.e., the hydroxide ion). In light of such considerations, as can be seen from the kinetic constant reported in Table 1, the nucleophilic attack is the RDS for both the hydrolysis mechanisms. Therefore, the corresponding free energy profiles were evaluated by means of the PMM approach (Figure 6).

In alkaline catalysis, the reactant, which featured a net charge separation, is strongly stabilized by the solvent. The effect is less pronounced in the transition state, resulting in an increase in the activation barrier with respect to the in-vacuum condition.

As reported in Table 2, the RDS rate constant is significantly higher in the alkaline catalysis (Suc → Gem step) with respect to the water-mediated mechanism, suggesting that the succinimide hydrolysis proceeds mainly via alkaline hydrolysis, even at neutral pH. Such a hypothesis is fully consistent with the strong correlation observed between deamidation rates and pH values, where a slope of ≃1 in a log(*k*) vs pH plot above pH 6 was observed.^50,51^

**Table 2:** Free Energy and Kinetic Rate Constants for Suc → Gem Transformation (300 K and pH = 7)

	Δ*A*^‡^ (kcal/mol)	*k*_L_ (s^–^^1^)	*k*_E_ (s^–^^1^)
water-mediated	31.2	2.7 × 10^–^^14^	1.2 × 10^–^^10^
alkaline catalysis	5.3	2.0 × 10^2^	9.2 × 10

### 4.3 Overall Deamidation Process

In light of the findings discussed above, the formation of succinimide and its hydrolysis need to be taken into account to evaluate the deamidation reaction kinetics. The kinetic rate constants and the activation free energy values for the overall reaction Asn^–^ → Gem (see data in Table 3) suggest that the deamidation kinetic is, at least in the studied system, mainly determined by the succinimide formation.

**Table 3:** Calculated Activation Free Energies and Kinetic Rate Constants for the Sub-Reactions Needed to Transform Asn^–^ in Gem at 300 K and Neutral pH^a^

reaction step	Δ*A*^‡^ (kcal/mol)	*k*_E_ (s^–^^1^)	*k*_L_ (s^–^^1^)
Asn^–^→ Tet^–^	18.7	1.40 × 10^–^^1^	7.0 × 10^–^^1^
Tet^–^→ Asn^–^	9.73	5.12 × 10^5^	4.3 × 10^6^
Tet^–^→ Suc + NH^–^_2_	16.5	5.79	28.9
Suc → Gem	5.26	9.20 × 10	2.0 × 10^2^

a The estimated statistical error is within 0.5% for Δ*A*^‡^, while it is under 1% for *k*_L_ and 4% for *k*_E_.

To validate these results, the calculated kinetic data were compared with the experimental results available in the literature. First of all, as no data are available for our model system, we considered experimental data on oligopeptides containing the Asn–Gly sequence. Among the several experimental works focused on deamidation kinetics, some are centered on the kinetics of succinimide formation and/or its hydrolysis,^11,23,50,52^ while others refer to the overall deamidation process, following the disappearance (onset) of Asn (Asp).^2,53^ Notably, regarding the succinimide hydrolysis kinetics, we cannot compare our results to the experimental ones due to the two parallel competitive reactions that occur (see Figure 1) under experimental conditions. However, Xie and co-workers results on succinimide hydrolysis mechanism are in good agreement with our alkaline catalysis hypothesis at neutral pH.^51^

To the best of our knowledge, there are no experimentally determined *k*_1_, k_–1_, and *k*_2_; thus, we could only compare deamidation half-life times, as reported in Table 4.

**Table 4:** Comparison of Experimental and Calculated Deamidation τ_1/2_, Obtained by the Propagation of eqs 18–20^a^

τ_1/2_ (days)	Calc_L_	Calc_E_	Exp	Exp	Exp	Exp	Exp
	1.70	5.07	1.41^b^	1.03^c^	4.96^d^	1.89^e^	1.14^f^

a Calc_L_ (Calc_E_) is the reaction half-life times considering Landau (Eyring)-derived rate constants; its statistical error never exceeds 3% (5%). All the experimental results were obtained at 310 K and neutral pH (7.4–7.5) with TrisHCl as buffer, unless 4.96^11^ and 1.89^2^ extrapolated at zero buffer concentration and in phosphate buffer, respectively.^2,11^

b ref (23).

c ref (53).

d ref (11).

e ref (2).

f ref (54).

## 5 Conclusions

As a common post-translational modification (PTM), deamidation holds significant importance in the development of biologics. This chemical process, involving the conversion of asparagine or glutamine residues to aspartic acid or glutamic acid, respectively, can occur spontaneously under various physiological conditions.^55^ Deamidation can profoundly impact the stability, efficacy, and immunogenicity of therapeutic proteins, making it a critical consideration in biopharmaceutical development. To understand and mitigate the effects of deamidation, several computational approaches were proposed for estimating the rates and exploring the underlying mechanisms of this PTM.^10,13−15,48^

In this work, we presented a theoretical-computational approach to reconstruct the overall deamidation kinetics in solution, evaluating the kinetic rate constants of the three reaction steps, e.g., ring closure, deamination, and hydrolysis. From the explicit treatment of these stages in solution, it comes out that the Tet^–^ → Asn^–^ backward reaction plays a central role in deamidation kinetics. In fact, the rate of succinimide formation, which accounts for the overall deamidation process, is strictly affected by *k*_1_ and *k*_–1_. Notably, the forward and backward ring-closure reactions are often neglected by approximating the succinimide formation to a single irreversible step. Our data, in line with available experimental data evaluated in similar systems (i.e., short peptides), represent the first step toward the possibility to predict the PTM rate in different contexts, i.e., proteins, by theoretical–computational procedures based on an accurate treatment of the environment.

## Supporting Information Available

Absolute energies, imaginary frequencies, and geometries of stationary points in gas phase; population profiles of the species involved in succinimide formation applying the two consecutive irreversible reaction kinetic scheme; bidimensional in-vacuum potential energy surface of Gem to Asp subreaction within the water-mediated mechanism. (PDF)
