# Supplementary material for: Molecular Modeling of the Deamidation Reaction in Solution: A Theoretical–Computational Study
Source: J Phys Chem B. 2023 Oct 30;127(44):9550–9. doi: 10.1021/acs.jpcb.3c04662 (PMC10641835; doi:10.1021/acs.jpcb.3c04662)
Supplement: Supplementary file 1 — jp3c04662_si_001.pdf [file jp3c04662_si_001.pdf]

Molecular Modeling of the Deamidation Reaction in Solution: a Theoretical-Computational Study

Maria Laura De Sciscio,[†] Alessandro Nicola Nardi,[†] Fabio Centola,[‡] Mara Rossi,[‡] Enrico Guarnera,[*,‡,¶] and Marco D'Abramo[*,†]

†*Department of Chemistry, University of Rome, Sapienza, P.le A. Moro 5, 00185 Rome, Italy*

‡*Global Analytical Pharmaceutical Science and Innovation, Merck Serono S.p.A., Rome, Italy*

¶*Antibody Discovery & Protein Engineering, Merck KGaA, Darmstadt, Germany*

E-mail: enrico.guarnera@merckgroup.com; marco.dabramo@uniroma1.it

Table S1: Gas phase properties of the deamidation sub-steps stationary points.

	Energy (a.u.)	Imaginary Frequencies (cm^{-1})
Succinimide formation		
Asn^-	-511.5118969	None
TS	511.480464	-145.6
Tet^-	-511.4814802	None
TS^*	-511.4472978	-119.0
Alkaline Hydrolysis		
Suc + OH^- + H_2O	-607.8507058	None
TS1	-607.8475490	-132.0
Gem	-607.8595553	None
TS2	-607.8468653	-118.4
Int	-607.8630223	None
TS3	-607.8621620	-542.7
Asp	-607.8984468	None
Water-mediated Hydrolysis		
Suc + 2 H_2O	-608.4268527	None
TS1	-608.3675898	-1134.3
Gem	-608.3996768	None
TS2	-608.3614966	-229.0
Asp	-608.4113306	None

* is Suc + NH_2^-, i.e. the last species considered in the deamination subprocess, due to the instability of NH_2^- in gas phase.

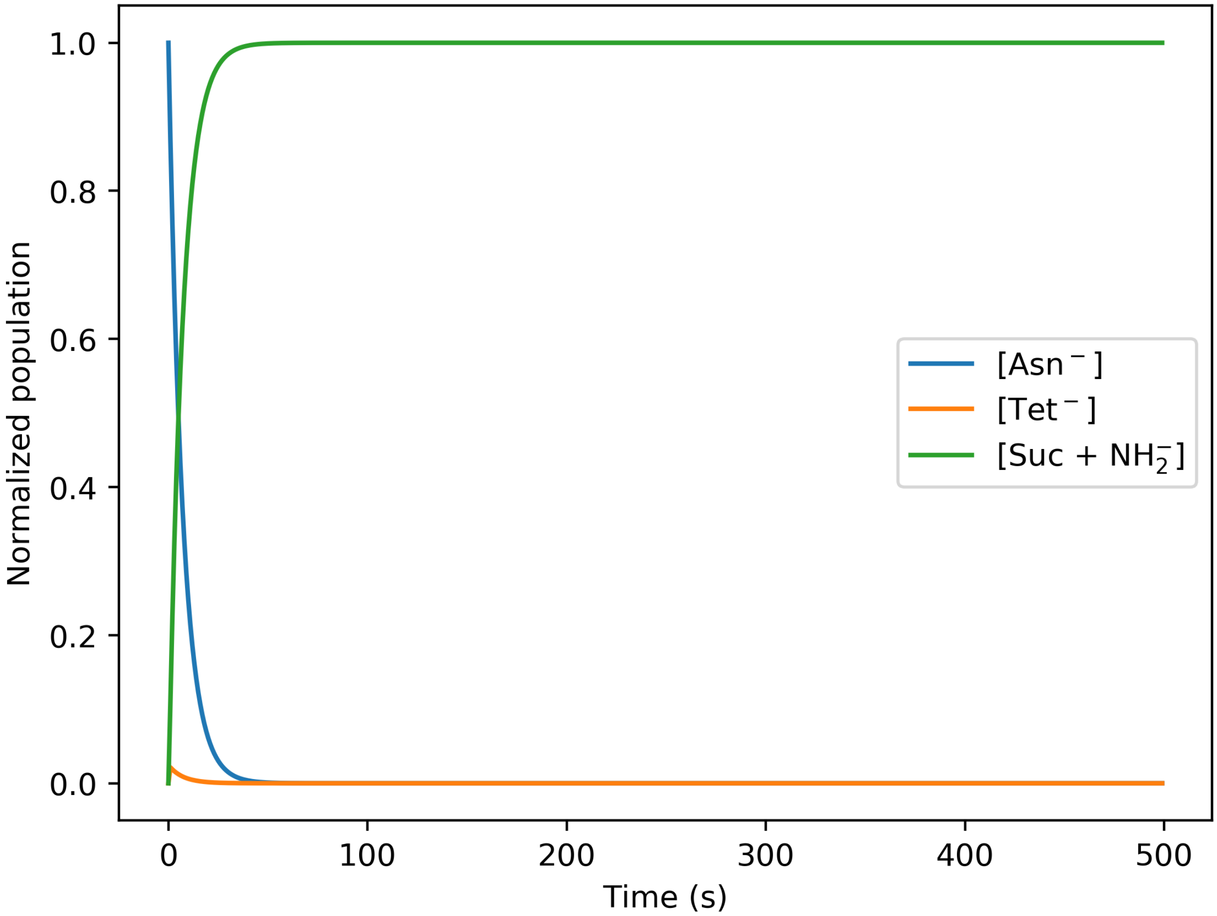

Figure S1: Time evolution of Asn^-, Tet^- and $Suc+NH_2^-$ concentrations within the irreversible kinetic model; $k_{E1} = 1.40 \cdot 10^{-1}$ and $k_{E2} = 5.79$ s^{-1}.

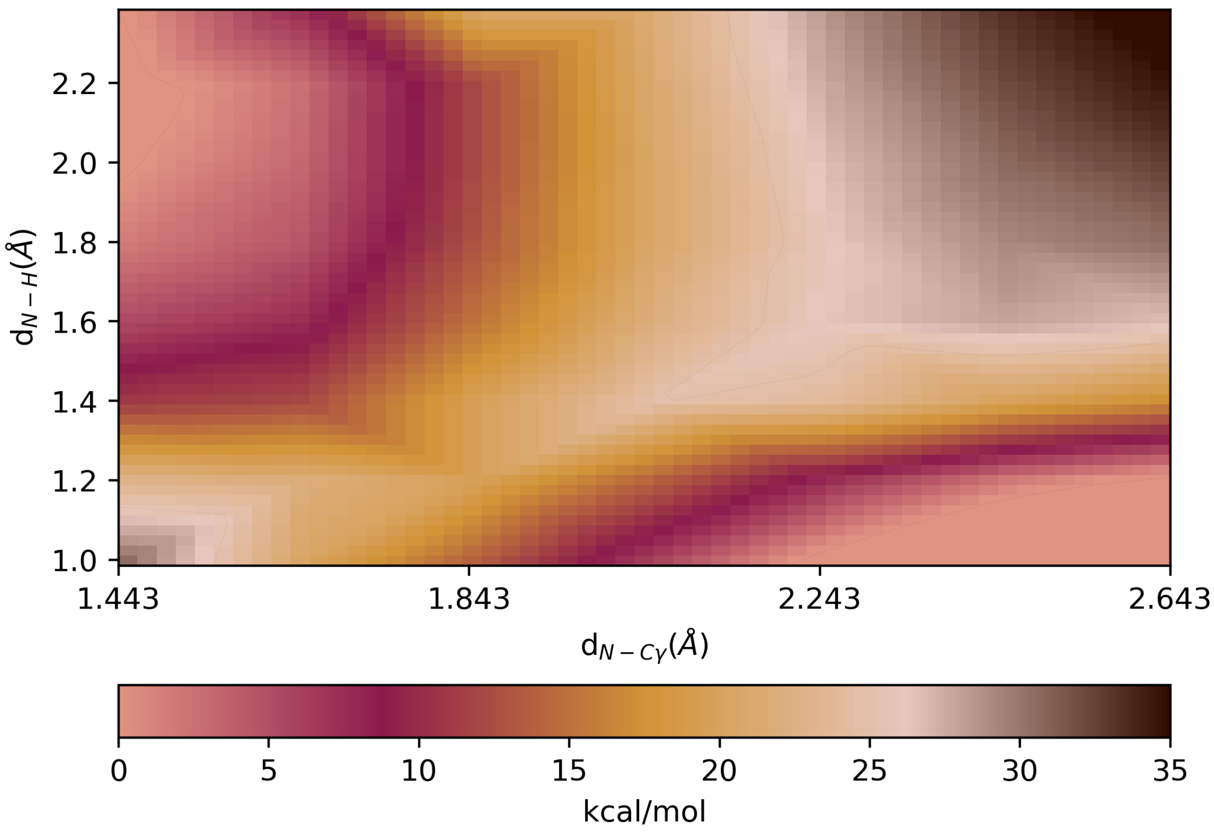

Figure S2: Bidimensional gas-phase potential energy surface of Gem → Asp with two water molecules. i.e. water-mediated mechanism.
